# A Small Genomic Region Containing Several Loci Required for Gastrulation in *Drosophila*

**DOI:** 10.1371/journal.pone.0007437

**Published:** 2009-10-13

**Authors:** Sam J. Mathew, Stephen Kerridge, Maria Leptin

**Affiliations:** 1 Institute of Genetics, University of Cologne, Cologne, Germany; 2 IBDML-UMR6216, Case 907 Parc Scientifique de Luminy, Marseille, France; Fred Hutchinson Cancer Research Center, United States of America

## Abstract

Genetic screens in *Drosophila* designed to search for loci involved in gastrulation have identified four regions of the genome that are required zygotically for the formation of the ventral furrow. For three of these, the genes responsible for the mutant phenotypes have been found. We now describe a genetic characterization of the fourth region, which encompasses the cytogenetic interval 24C3-25B, and the mapping of genes involved in gastrulation in this region. We have determined the precise breakpoints of several existing deficiencies and have generated new deficiencies. Our results show that the region contains at least three different loci associated with gastrulation effects. One maternal effect gene involved in ventral furrow formation maps at 24F but could not be identified. For a second maternal effect gene which is required for germ band extension, we identify a candidate gene, *CG31660*, which encodes a G protein coupled receptor. Finally, one gene acts zygotically in ventral furrow formation and we identify it as *Traf4*.

## Introduction

The process of gastrulation in *Drosophila* begins with the invagination of the mesoderm on the ventral side of the blastoderm stage embryo. The genes that determine the cell fates in this region are known and have been extensively studied, but the genes whose products are directly responsible for mediating the morphogenetic events such as the cell shape changes that create the ventral furrow and thereby internalize the mesodermal cells have remained more elusive. Three zygotically active genes affecting gastrulation, *twist, snail* and *folded gastrulation,* were found in classical genetic screens. The Twist and Snail proteins are essential for gastrulation: in their absence proper cell shape changes do not take place, and the mesodermal cells fail to invaginate. However, they are transcription factors, and as such cannot directly modulate cellular activities. The product of *folded gastrulation* is a secreted peptide that is believed to act through a G-protein coupled receptor, but its loss of function phenotype is weak, and it can therefore not be the only mediator of cell shape changes.

Two screens using large genomic deficiencies to identify missing zygotic loci involved in ventral furrow formation showed that in addition to the known genes, four further regions of the genome played a role in this process [1], [2]. Embryos that were homozygous deficient for these regions show delays and other defects during gastrulation. The genes in three of these regions have been identified and characterized [2], [3], [4], [5], [6].

The fourth region, encompassing the cytogenetic stretch 24C3-25A on the left arm of the second chromosome, has proved difficult to analyse. Genetic analyses are complicated by the fact that the region contains one of the largest genes in the *Drosophila* genome, *dumpy* [7], [8], alleles of which are also present on Balancer chromosomes, which means that deficiencies uncovering *dumpy* cannot be used in crosses with these balancers. In addition, an unidentified haplo-insufficient locus lies close to this cytogenetic region [9] necessitating the use of duplications to maintain stocks carrying deficiencies uncovering the region of interest. Efforts to map a dominant maternal effect gene involved in gastrulation, *accordion*, suggested that the locus maps at 24F1-24F4, but the gene itself could not be molecularly identified [10].

The complexity of the region and the possible presence of at least one gene involved in gastrulation therefore called for a thorough and precise analysis of this interval. The availability of new lethal transposon insertions [11], [12] as well as genetic tools for the creation of defined genomic deletions [13], [14], [15] and single embryo PCR have allowed us to characterize the genetics of this region at high resolution and to show that it contains at least three loci involved in gastrulation. We report the identification of two of the genes here.

## Results and Discussion

### Gastrulation defects in embryos deficient for the 24C3-25A cytogenetic region

**Figure 1:**
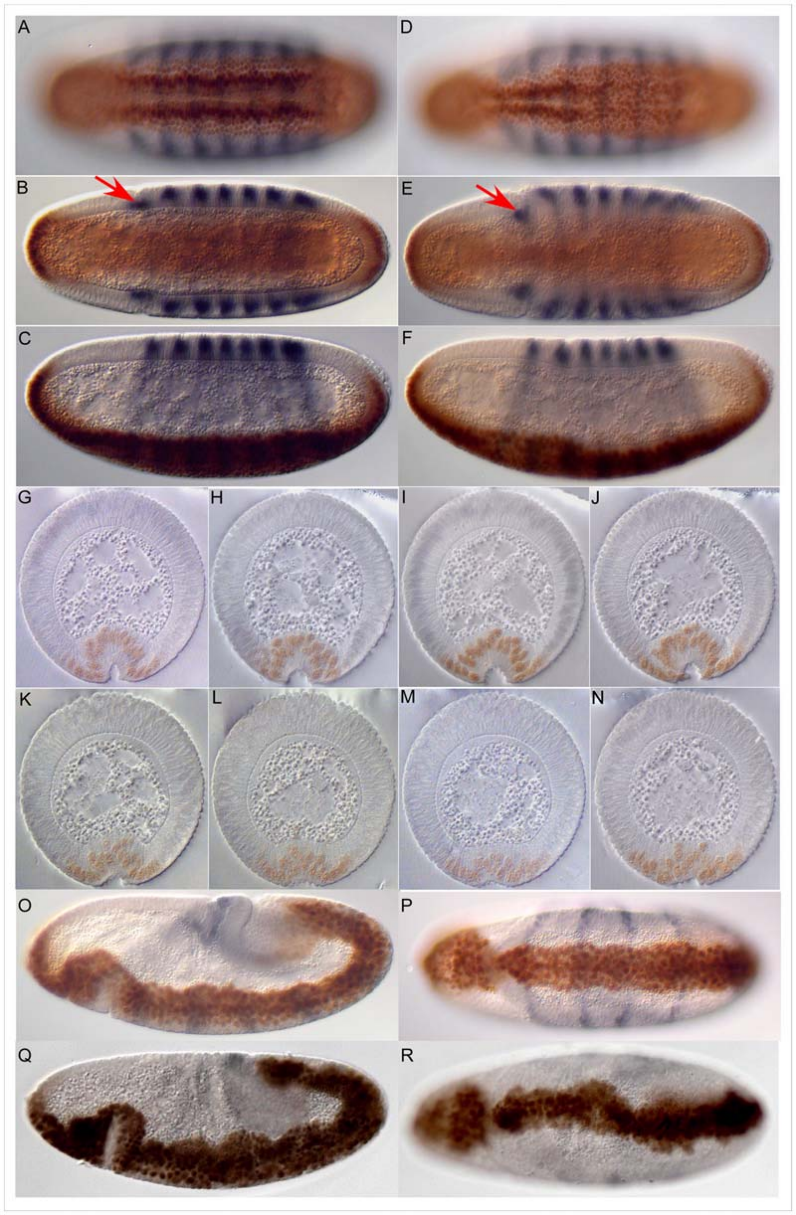
Defects in embryos lacking the 24C3-25A cytogenetic region. A–F. Wild type (A, B and C) and homozygous *Df(2L)ed-dp* (D, E and F) stage 5 embryos stained for Even-skipped (blue) and Twist (brown). Ventral (surface and mid-sagittal optical section) and lateral views are of the same embryo in each case. The mid-sagittal optical sections show the depth of the head fold (red arrow), which is used together with the shape of the Even-skipped stripes as a measure for the age of the embryo. G–N. Sections through wild type (G–J) and homozygous *Df(2L)ed-dp* (K–N) embryos stained for Even-skipped (blue) and Twist (brown) from the anterior of the embryo to the posterior. O–R. Later defects. Lateral and ventral views of wild type (O, P) and homozygous *Df(2L)ed-dp* (Q, R) stage 5 embryos stained for Even-skipped (blue) and Twist (brown).

One of the deficiency stocks uncovering genomic material in the 24C3-25A cytogenetic region used in the original screen, which exhibited gastrulation defects was *Df(2L)ed-dp* [2], [16]. Collections of embryos from heterozygous *Df(2L)ed-dp/SM1* parents stained with antibodies against Twist [17] and Even-skipped [18] show a range of defects. As previously described [2], [16] we used the depth of the head fold, the positions of the Eve-stripes, the appearance of dorsal folds and the position of the pole cells as markers for developmental time in our comparison of wild type and mutant embryos. About a quarter of the embryos from the *Df(2L)ed-dp/SM1* stock exhibit a delay in the invagination of the ventral furrow as compared to wild type embryos (Figure 1A–N). The mutant embryo shown in Figure 1D–F is slightly older by these criteria than the wild type embryo in Figure 1A–C, but the mesoderm has not yet invaginated fully. The asynchrony of invagination along the anterior-posterior axis exhibited by this embryo is frequently seen and is also evident in serial sections from the anterior end to the posterior end of mutant embryos (Figure 1K–N). These images show that cell shape changes in the mesoderm can occur, but do so in an uncoordinated or inefficient manner.

At slightly later stages, defects in spreading of the mesoderm on the underlying ectoderm can be seen (Figure 1O–R), but eventually the mesoderm is fully internalized in all embryos. The defects we observe at successive stages of gastrulation could be the result of the failure of different genes, each acting at a different stage, or the late defects could be due to the early failure in ventral furrow formation. Mapping of the locus or loci causing the defects should resolve this question.

### Deficiencies in the 24C-25A cytogenetic region

**Figure 2:**
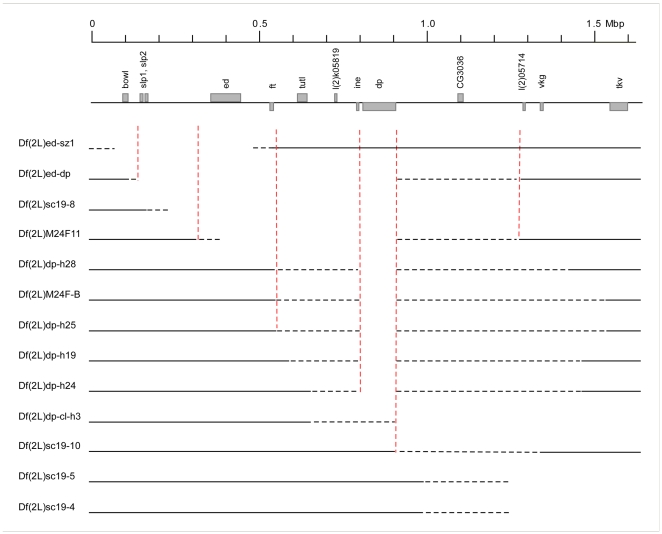
A map of the cytogenetic region 24C-25E, representing the classical deficiencies used in this study. The region is shown as a line with some of the landmark genes marked as grey boxes. The numbers between the genes indicate the numbers of known and predicted genes in the interval. Each deficiency is shown as a line, with the solid line denoting the region that is not affected, dotted lines indicating regions of uncertainty and the blank space indicating regions known to be deleted either by genetic or molecular mapping. The scale bar above shows Mega base pairs. The vertical, dotted red lines are for orientation.

To map the locus or loci responsible for the phenotypes described above we began by analysing all of the available deficiencies, which uncover genomic stretches mapping to this region to which we will refer as the “classical deficiencies” (Figure 2 and see Table S1 for a full list). We did not work with all available deficiencies because some stocks gave variable genetic results or were not sufficiently healthy for our phenotypic analyses.

A major drawback of the classical deficiencies is the imprecise mapping of their breakpoints, which is often based on complementation data with alleles of only a few genes. Figure 2 shows thirteen classical deficiencies used in this study with the ambiguous regions represented by dotted lines. We therefore mapped the breakpoints of the deficiencies more precisely both genetically by complementation analysis and molecularly by single embryo PCR. For the complementation analysis we used lethal alleles of all available mutants for genes in the region. For single embryo PCR we used primer sets for genes around the suspected break points (for a full list of the genes and respective primer sets used, see Table S2). We collected individual stage 16 or older embryos from egg collections of the stocks and assayed between 15 and 20 embryos per genotype. If one quarter of the tested embryos did not show a PCR product for a gene (but did show products for control genes elsewhere in the genome) we concluded that the gene was uncovered by the deficiency. Examples for *Df(2L)dp-h24* and *Df(2L)dp-h28* are shown in Figure S1.

The results from the analyses are summarised in Table 1. In three cases we found that genes were uncovered genetically, but the DNA assayed by PCR was still present (*dumpy* over *Df(2L)dp-h28* and *Df(2L)dp-h24*, and *Tps1* over *Df(2L)dp-h19*), suggesting that the breakpoint lies within the gene, or lies in the vicinity of the gene and affects its expression.

**Table 1:** Mapping of breakpoints of deficiencies in the 24C3-25A cytogenetic region.

Gene	method	M24	h28	h25	h19	h24	ED 252	ED 250	ED 251	Exel 6010	Exel 9062	Exel 3006
CG3714	comp	**+++**	**+++**	**+++**	**+++**	**+++**	**+**	**+**	**+**	**+**	**+**	**+**
***ft***	comp	**+++**	**+++**	**+++**	**+++**	**+++**	**+**	**+**	**+**	**+**	**+**	**+**
CG3702		**?**	**?**	**+**	**+**	**+**	**+**	**+**	**+**	**+**	**+**	**+**
RpL40		**?**	**?**	**+**	**+**	**+**	**+**	**+**	**+**	**+**	**+**	**+**
CG15425		**?**	**?**	**+**	**+**	**+**	**+**	**+**	**+**	**+**	**+**	**+**
CG3675		**?**	**?**	**+**	**+**	**+**	**+**	**+**	**+**	**+**	**+**	**+**
***tutl***	comp	**−**	**−**	**+++**	**+++**	**+++**	**+**	**+**	**+**	**+**	**+**	**+**
CG16857				**+**	**+**	**+**	**+**	**+**	**+**	**+**	**+**	**+**
Atet	PCR		**−**	**+++**	**+++**	**+**	**+**	**+**	**+**	**+**	**+**	**+**
CG15429	PCR		**−**	**−**	**+++**	**+**	**+**	**+**	**+**	**+**	**+**	**+**
CG15430					**+**	**+**	**+**	**+**	**+**	**+**	**+**	**+**
Traf4	PCR		**−**	**−**	**+++**	**+**	**+**	**+**	**+**	**+**	**+**	**+**
CG17612					**+**	**+**	**+**	**+**	**+**	**+**	**+**	**+**
CG3338					**+**	**+**	**+**	**+**	**+**	**+**	**+**	**+**
CG18013	comp	**−**	**−**	**−**	**+++**	**+++**	**+**	**+**	**+**	**+**	**+**	**+**
Tps1	PCR		**−**	**−**	**+++**	**+++**	**+**	**+**	**+**	**+**	**+**	**+**
Tps1	comp	**−**	**−**	**−**	**−**	**+++**	**+**	**+**	**+**	**+**	**+**	**+**
CG3652	PCR	**−**	**−**	**−**	**−**	**+++**	**+**	**+**	**+**	**+**	**+**	**+**
***l(2)k05819***						**+**	**+**	**+**	**+**	**+**	**+**	**+**
CG3058						**+**	**+**	**+**	**+**	**+**	**+**	**+**
CG15431						**+**	**+**	**+**	**+**	**+**	**+**	**+**
CG12677	PCR		**−**	**−**	**−**	**+++**	**+**	**+**	**+**	**+**	**+**	**+**
CG15432						**?**	**+**	**+**	**+**	**+**	**+**	**+**
morgue						**?**	**+**	**+**	**+**	**+**	**+**	**+**
CG15433						**?**	**+**	**+**	**+**	**+**	**+**	**+**
CG15438						**?**	**+**	**+**	**+**	**+**	**+**	**+**
CG15439						**?**	**+**	**+**	**+**	**+**	**+**	**+**
	5-HA-1707					**?**		**+**	**+**	**+**	**+**	**+**
CG15440						**?**		**+**	**+**	**+**	**+**	**+**
CG15434						**?**		**+**	**+**	**+**	**+**	**+**
Gs1l						**?**		**+**	**+**	**+**	**+**	**+**
RpL27A	comp	**−**	**−**	**−**	**−**	**−**		**+**	**+**	**+**	**+**	**+**
CG15435								**+**	**+**	**+**	**+**	**+**
CG15443								**+**	**+**	**+**	**+**	**+**
CG15436	PCR	**−**	**−**	**−**	**−**	**−**		**+**	**+**	**+**	**+**	**+**
CG17840			**?**			**?**		**+**	**+**	**+**	**+**	**+**
***ine***			**?**			**?**		**+**	**+**	**+**	**+**	**+**
	CB-5668-3		**?**			**?**				**+**	**+**	**+**
***dp***	comp	**−**	**−**	**−**	**−**	**−**				**+**	**+**	**+**
***dp***	PCR	**−**	**+++**	**−**	**−**	**+++**				**+**	**+**	**+**
CG15636			**+**			**+**				**+**	**+**	**+**
CG11934			**+**			**+**				**+**	**+**	**+**
CG11933			**+**			**+**				**+**	**+**	**+**
CG11932			**+**			**+**				**+**	**+**	**+**
CG15635			**+**			**+**				**+**	**+**	**+**
CG11931			**+**			**+**				**+**	**+**	**+**
CG3355			**+**			**+**				**+**	**+**	**+**
CG11930			**+**			**+**				**+**	**+**	**+**
CG11929	PCR	**−**	**+++**	**−**	**−**	**+++**				**+**	**+**	**+**
Bsg25A			**+**			**+**				**+**	**+**	**+**
CG15634	PCR	**−**	**+++**	**−**	**−**	**+++**				**+**	**+**	**+**
CG3251			**+**			**+**				**+**	**+**	**+**
Taf30a-2			**+**			**+**				**+**	**+**	**+**
CG15633			**+**			**+**				**+**	**+**	**+**
CG11928			**+**			**+**				**+**	**+**	**+**
CG15631	PCR	**−**	**−**	**−**	**−**	**−**				**+**	**+**	**+**
CG15630		**?**	**+**		**?**	**+**				**+**	**+**	**+**
CG3294		**?**	**+**		**?**	**+**				**+**	**+**	**+**
CG3244		**?**	**+**		**?**	**+**				**+**	**+**	**+**
CG15629		**?**	**+**		**?**	**+**				**+**	**+**	**+**
CG3225	PCR	**+++**	**+++**	**−**	**+++**	**+++**				**+**	**+**	**+**
	5-HA-1531	**+**	**+**	**?**	**+**	**+**					**+**	**+**
CG15628		**+**	**+**	**?**	**+**	**+**		**+**			**+**	**+**
CG2976		**+**	**+**	**?**	**+**	**+**		**+**			**+**	**+**
CG15627		**+**	**+**	**?**	**+**	**+**		**+**			**+**	**+**
CG15626		**+**	**+**	**?**	**+**	**+**		**+**			**+**	**+**
CG12194		**+**	**+**	**?**	**+**	**+**		**+**			**+**	
CG2950		**+**	**+**	**?**	**+**	**+**		**+**			**+**	
CG11927	comp	**+**	**+**	**?**	**+**	**+**		**+**		**−**	**−**	**−**
mRpS2	comp	**+++**	**+++**	**+++**	**+++**	**+++**		**+**		**−**	**−**	**−**
CG11926		**+**	**+**	**+**	**+**	**+**		**+**				
CG31660		**+**	**+**	**+**	**+**	**+**		**+**				
Cf2		**+**	**+**	**+**	**+**	**+**		**+**				**+**
CG3008		**+**	**+**	**+**	**+**	**+**		**+**				**+**
CG15625		**+**	**+**	**+**	**+**	**+**		**+**		**+**	**+**	**+**
	5-HA-1621 CB-0110-3	**+**	**+**	**+**	**+**	**+**		**+**		**+**	**+**	**+**
***CG3036***		**+**	**+**	**+**	**+**	**+**	**+**	**+**	**+**	**+**	**+**	**+**

The left column lists all predicted and known genes in the region. The second column indicates which genes were tested and by what method. It also indicates the elements used for generating new deficiencies. The method for testing the classical deficiencies was either single embryo PCR (PCR) or complementation (comp). The deficiencies are abbreviated; please refer to Table S1 for the full names. A ‘+++’ sign indicates that the gene is not uncovered, a ‘—’ that it is uncovered. Areas marked by ‘+’ are the extrapolations from the experimental data for regions that are not deleted, blank fields to regions that are deleted. For the Drosdel and Exelixis deletions these regions are deduced from the P-element insertions that were used to generate the deficiencies. Regions of uncertainty are denoted by ‘?’. Genes highlighted in bold italic are those that are listed at the top of Figure 2 for orientation.

Note that in *Df(2L)dph28* and *Df(2L)dph24* we obtain no PCR product for *CG15631*, although the primers work well on wild type flies (see Figure S1).

We do not understand this result, but assume that either the parent stock from which both deficiencies were derived had a mutation that affects this gene, or in each case a small lesion was generated during the rearrangement that created the deficiencies.

**Figure 3:**
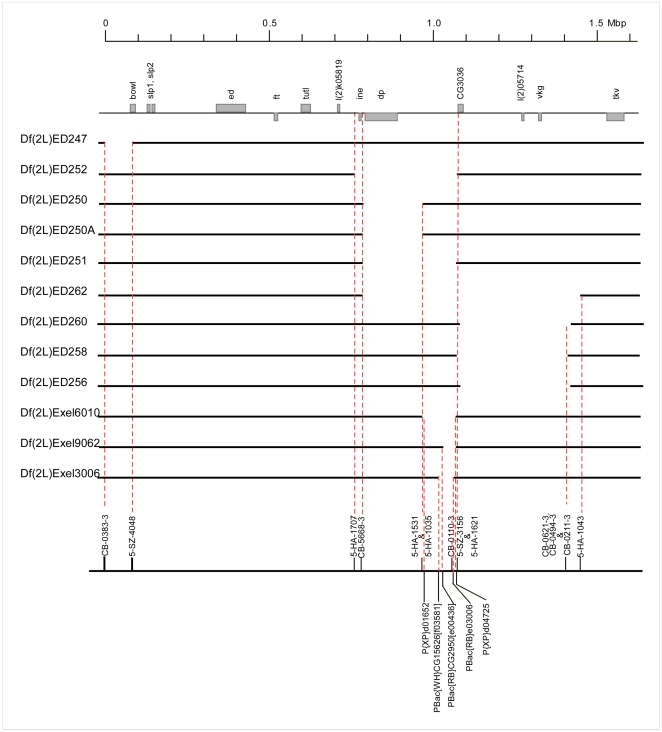
Newly generated deficiencies. Same representation as in Figure 2. In addition, the forward and reverse elements used to generate the deletions are shown at the bottom. The figure lists ten newly generated deficiencies, nine using Drosdel insertions (Df(2L)ED247 to Df(2L)ED256) and one (Df(2L)Exel3006) generated with forward and reverse elements from the Exelixis Consortium. In addition, two deficiencies obtained from the Exelixis collection (Df(2L)Exel6010 and Df(2L)Exel9062) are also shown.

For additional resolution and certainty of genetic mapping within the region, we used stocks from the Drosdel and Exelixis consortia [12], [14] to generate ten new deficiencies with precisely defined breakpoints between 24A and 26A (Figure 3).

### Mapping of loci involved in the control of gastrulation

In order to narrow down the genetic interval containing the gene or genes responsible for the gastrulation defects seen in embryos from the *Df(2L)ed-dp/SM1* stock, other, overlapping-deficiencies were evaluated. It was not possible to analyse embryos that were homozygous for the deficiencies in all cases, because many of the deficiencies (all of the *sc19* deficiencies as well as *Df(2L)dp-cl-h3*) are kept in balanced stocks carrying duplications of the deleted region. Consistent with this, embryos that were collected from the *Df(2L)dp-cl-h3* stock, for example, looked nearly normal, with only slight early defects. Some deficiencies were therefore tested by crossing males from the stocks to females that were heterozygous for the deficiencies *Df(2L)ed-dp* or *Df(2L)dp-h19*. One eighth of the progeny of such crosses are transheterozygous for the deficiencies and do not carry the duplication. The phenotypes in many of the crosses appeared with varying penetrance and expressivity. Attempts to quantify them were abandoned, because of the variability in the general healthiness of the stocks, in the number of eggs laid and fertilized and other parameters that made an absolute and quantifiable evaluation impossible. Instead, several collections from each cross were examined in a double-blind manner by three experienced observers (ML, SM and Thomas Seher) and phenotypic defects scored as strong, medium, weak or absent. Table 2 summarizes the crosses performed and the phenotypes observed.

**Table 2:** Phenotypes of embryos heterozygous for deficiencies.

♀	ed-sz1		ed-dp		dp-h28		M24F-B		dp-h25		dp-h19		dp-h24		dp-cl-h3	
♂	vf	late	vf	late	vf	late	vf	late	vf	late	vf	late	vf	late	vf	late
ed-sz1	−	−														
sc19-8			++	+												
ed-dp			+++	+++											(+)	−
M24F-11			+++	+++												
dp-h28					+	+										
M24F-B							++	++								
dp-h25									++	+++*						
dp-h19			+++	+							+++	+++			(+)	−
dp-h24													−	−		
dp-cl-h3															(+)	−
sc19-10											++	++				
sc19-5			++	+							+++	++				
sc19-4			++	+												
wt			++	+							++	+				

Males (columns) and females (rows) from the indicated stocks were crossed to each other and embryos from the crosses were stained with antibodies against Twist and Even-skipped. They were evaluated independently by three observers for the defects in ventral furrow formation and later events in gastrulation as described in the text. Defects were scored as very strong (+++) to weak ((+)) or absent (−). If a field does not contain a value, the cross was not performed.

*Embryos from this stock show a strong general patterning defect.

The main outcome can be summarized as follows. *Df(2L)ed-sz1* embryos appeared normal, indicating that the region distal to the *fat* gene need not be considered further.

The full range of phenotypes initially observed for *Df(2L)ed-dp* (delayed ventral furrow invagination, mesoderm spreading defects and malformed germ band) was also exhibited by embryos derived from the stocks carrying deficiencies *Df(2L)dp-h19* and *Df(2L)dp-h28*, although in the latter they were generally weaker.

The late defects showed different complementation behaviours than the defects in ventral furrow formation. They are seen in transheterozygotes of *Df(2L)ed-dp* over *Df(2L)sc19-8* or over *Df(2L)M24F-11*, but were much weaker in *Df(2L)ed-dp* over *Df(2L)dp-h19* transheterozygotes, although each deficiency in homozygosity resulted in the full range of phenotypes. We assume that each of the deficiency stocks may have accumulated independent background mutations elsewhere in the genome, which, perhaps in conjunction with the delay in the formation of the ventral furrow, lead to late gastrulation defects. This is not unlikely, as defects such as twisted germ band are often observed in weak stocks. It is also consistent with observations that the *Df(2L)dp-h19* stock failed to complement lethal alleles from outside the 24C3-25A region during crosses performed during other, unrelated experiments (data not shown). Therefore, although the late phenotypes are potentially interesting, we primarily concentrated on the early ventral furrow defects.

The defects in ventral furrow formation were seen in most of the crosses, except in those with the smallest deficiency, *Df(2L)dp-h24*, indicating that genes outside this region contribute to the observed defects. The phenotype was also detectable when females were crossed against wild type males, which shows that the phenotype is due at least in part to a dominant maternal effect. The smallest region or regions that might contain loci involved in ventral furrow formation is defined by the distal breakpoint of *Df(2L)dp-h25* (between *Atet* and *CG15429*) and the proximal breakpoint of *Df(2L)dp-h28* (within *dumpy*) (Table 1). We did not consider the narrower interval defined by the distal breakpoint of *Df(2L)dp-h19* as reliable in view of the inconsistent genetic behaviour described above.

Overall, the results from this complementation analysis cannot easily be explained by assuming that only one locus is responsible for the observed defects. We therefore carried out further analyses with the possibility in mind that two or more loci, acting either maternally or zygotically, and possibly in a dominant manner, might be involved.

### A maternal locus contributes to the gastrulation defects of embryos lacking the 24C – 25A cytogenetic region

To define the region or regions required for proper gastrulation more precisely, we analysed our newly generated smaller deficiencies for their phenotypes. Embryos from stocks carrying the deficiencies *Df(2L)ED250* or *Df(2L)ED250A* showed no gastrulation defects, consistent with the notion that a locus required for gastrulation maps distal to *dumpy*. This narrows the region in which the responsible gene or genes must lie to the interval between *Atet* at the distal end and *ine* at the proximal end.

Since the defects we observed were consistent with being caused by a dominant maternal effect, and since a dominant maternal effect locus, *Accordion*, had previously been described to map in the region 24F1-24F4, we tested alleles of genes in this region for their gastrulation phenotypes. Embryos homozygous for the lethal allele *l(2)SH0805* of the gene *CG18013*, for the *turtle* alleles *jf4^b11^* or *tutl^k14703^* and for the *jf6sz3* allele of the gene *l(2)24Fa* showed no gastrulation defects. We also tested 6 alleles of *Tps1* (one P element insertion and 5 EMS induced alleles of the *jf5* group– *jf5^a18^*, *jf5^a19^*, *jf5^b2^*, *jf5^h10^* and *jf5^sz31^*). We found that one mutant allele *l(2)jf5^sz31^* or *Tps1^sz31^* [19], showed a dominant maternal effect on gastrulation. However, none of the other five available alleles of this gene showed this phenotype. Furthermore, we found that *Tps1^sz31^* also fails to complement alleles of *dumpy*, and down regulates the expression of the nearby *Traf4* gene in the mesoderm (not shown) indicating that there are additional lesions nearby. We have not analysed these further, but the findings support the notion of the existence of a dominant maternal effect gene with a role in gastrulation in this region.

### A maternal effect gene involved in germ band extension

**Figure 4:**
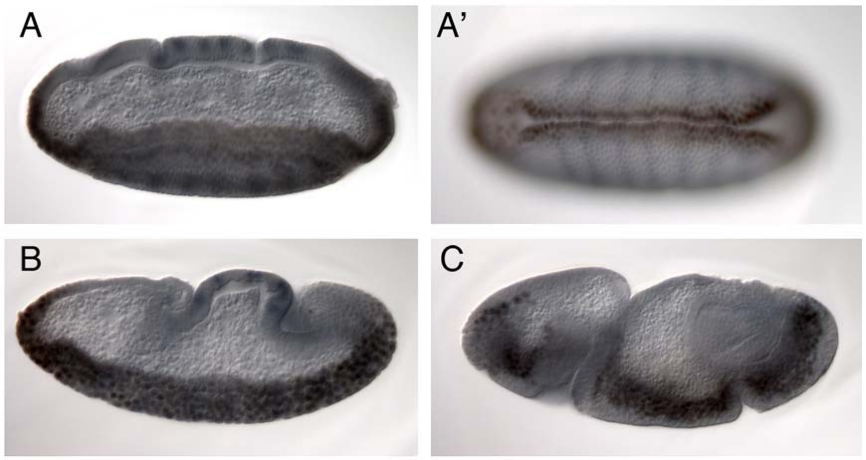
Embryos from *pog* mutant germ line clones. A, A′. Lateral and ventral views of a stage 5 embryo stained for Twist (brown) and Even skipped (blue). The ventral furrow has formed normally. An abnormal dent on the dorsal side of the head region appears at this stage. B. During germ band extension, the dorsal epithelium does not fold up in the normal way and the posterior midgut invagination does not move anteriorly at the normal rate. C. At later stages the posterior midgut invagination is seen to have remained in the posterior region of the embryo, and folds in the germ band appear.

An EMS mutation, *pog* (*poor gastrulation*), isolated in a screen for genes affecting wing vein morphogenesis in combination with *Atrophin* heterozygotes (SK, data not shown), had been shown by large-scale deficiency mapping to be uncovered by *Df(2L)sc19-8* and was therefore of interest in the context of this study. Embryos derived from homozygous *pog* germ line clones show severe defects in germ band extension (Figure 4B, C), but no defects in ventral furrow formation (Figure 4A, A′). This gene is therefore unlikely to be responsible for the phenotype observed for the deficiencies. The responsible gene was nevertheless mapped more precisely. Hemizygotes of *pog* over deficiencies die as 3–7 day old adults and are sterile. The small number of embryos that can be obtained from *Df/pog* females resemble *pog* germ line clone embryos. We used these phenotypes to map *pog* relative to the deficiency breakpoints and found that it was uncovered by *Df(2L)ED251, Df(2L)Exel 9062*, *Df(2L)Exel6010* and *Df(2L)Exel3006* (Table 1 and, Figure 3) but not by *Df(2L)ED250*. These complementation tests leave four genes (*CG11927*, *mRpS2*, *CG11926* and *CG31660*) as putative candidates. As *pog* complements the lethal alleles *CG11927^c04401^* and *mRpS2^EY10086^*, which are lethal over the small deficiencies in this region, indicating that their lethality is associated with the insertions, this leaves two candidate genes. *CG31660*, but not *CG11926*, mRNA is detected in preblastoderm embryos (data not shown). Although it is not formally excluded that maternally supplied *CG11926* protein might be present, these findings indicate that *pog* disrupts the G protein coupled receptor encoded by *CG31660*. A detailed functional analysis of *pog* will be presented elsewhere.

### A zygotic locus contributes to the gastrulation defects of embryos lacking the 24C – 25A cytogenetic region

Our results so far have shown that the defects observed in the embryos we have analysed can be explained at least in part by maternal effects. However, one of the screens leading to the identification of the region as being involved in gastrulation was conducted in such a way that only zygotic effects would have been observed [1], [20]. We therefore tested which of the deficiencies caused defects in a situation in which maternal effects can be excluded. To distinguish possible zygotic gastrulation effects from the maternal effect, the mothers used for such a cross must have two wild type copies for the region in question, whereas the progeny should nevertheless be homozygous mutant. This can be achieved by using stocks with compound chromosomes in which two chromosome arms segregate together instead of being separated during meiosis [20], [21]. We used the second compound chromosome *C(2)v* for our experiments. The chromosome segregation pattern in the compound stock as well as the segregation pattern in crosses of *C(2)v* females with males that are heterozygous for deficiencies are depicted in Figure S2.

When we crossed *C(2)v* females with wild type males we observed a weak but reproducible delay in furrow formation. This can most likely be ascribed to the haploinsufficiency for *snail* during gastrulation, which results in a delay in ventral furrow formation [16], [22]. A similar phenotype was seen in embryos from crosses of *C(2)v* mothers to males that were heterozygous for *Df(2L)dp-h19* or *Df(2L)dp-h24* indicating that with regard to gastrulation, these chromosomes behaved like wild type chromosomes. By contrast, crosses with fathers from stocks carrying *Df(2L)ed-dp*, *Df(2L)dp-h25*, *Df(2L)dp-h28* or *Df(2L)M24F-B* showed more dramatic failures in furrow formation. The region defined by these deficiencies must therefore contain a zygotically active locus required for proper ventral furrow formation.

### Interacting loci

**Figure 5:**
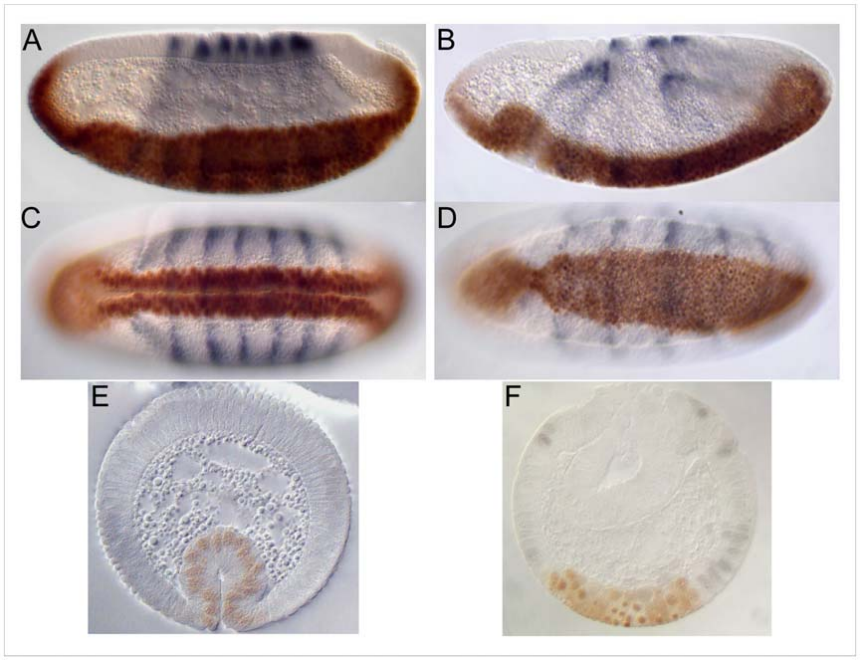
Phenotypes of embryos from crosses using the *C(2)v* chromosome. A–F. Lateral, ventral and section views of embryos stained for Twist (brown) and Even skipped (blue) from wild type (A, C and E), and *Df(2L)ed-dp* males crossed to *C(2)v* females (B, D and F) showing the severe delay in ventral furrow formation in the latter embryos.

The phenotype exhibited by embryos from crosses between males heterozygous for the deficiencies and *C(2)v* females was more severe than that of embryos from a cross between deficiency heterozygous males and females (Figure 5). This suggests that the aneuploidy created by the *C(2)v* chromosomes enhances the phenotype of the deficiency (note that this is not the case for all deficiencies, i.e. it is not a general property of crosses involving *C(2)v* females). Hemizygosity of all or part of the left arm of the second chromosome, or triploidy of part, or all of, the right arm, or both of these together might be responsible for this effect (See Figure S3 B, grey column). We attempted to determine the cause of the enhancement by two strategies, namely testing candidate genes and using partial deletions and duplications of the chromosome arms to recreate the effect of *C(2)v* and thereby map the responsible region. Both approaches were unsuccessful. We first created stocks yielding embryos that in addition to being homozygous for *Df(2L)ed-dp* were also heterozygous for *snail* or carried three copies of *twist* (Figure S3 A), but none of these showed significantly stronger phenotypes than the *Df(2L)ed-dp* homozygotes. A different way to test whether the effects were caused by three copies of *twist* was to reduce the dose of *twist* in the *C(2)v* crosses back to two copies by making recombinants between *Df(2L)ed-dp* and *twist* in these crosses. However, when males from such recombinant stocks were crossed to *C(2)v* females the phenotype was as strong as when the deficiency alone was used.

We then used translocation stocks to reproduce hemizygosity or triploidy of parts of the second chromosome in the background of deficiencies in order to map the region that interacts with the deficiency (Table S1 and, crossing scheme Figure S3). In no case did we reproduce the phenotype seen in the *C(2)v* crosses. The most proximal regions on each arm (cytogenetic region 39–40 on the left, cytogenetic region 41–43 on the right) could not be assessed in this manner as no translocations could be obtained for this stretch.

We had previously tested genetic interactions between loci involved in gastrulation, and found enhancement only between the deficiency uncovering the gene *t48* with *fog*, but none between the deficiencies described here and other known gastrulation loci [16], excluding the possibility that those might be responsible for the effects described here.

In summary, the enhancement is either due to a synergistic effect of hemizygosity of the left arm of the second chromosome and triploidy of the right arm of the second chromosome, or the regions not accessed by the translocation stocks might harbour the locus responsible for the enhancement.

### Mapping of the zygotic locus

The crosses of deficiency males against *C(2)v* females described above indicate that the locus responsible for the zygotic gastrulation phenotype lies between the distal breakpoint of the deficiency *Df(2L)dp-h19* and the distal breakpoint of *Df(2L)dp-h25* (Figure 2 and Table 1), and therefore between the genes *Atet* and *Tps1*.

**Figure 6:**
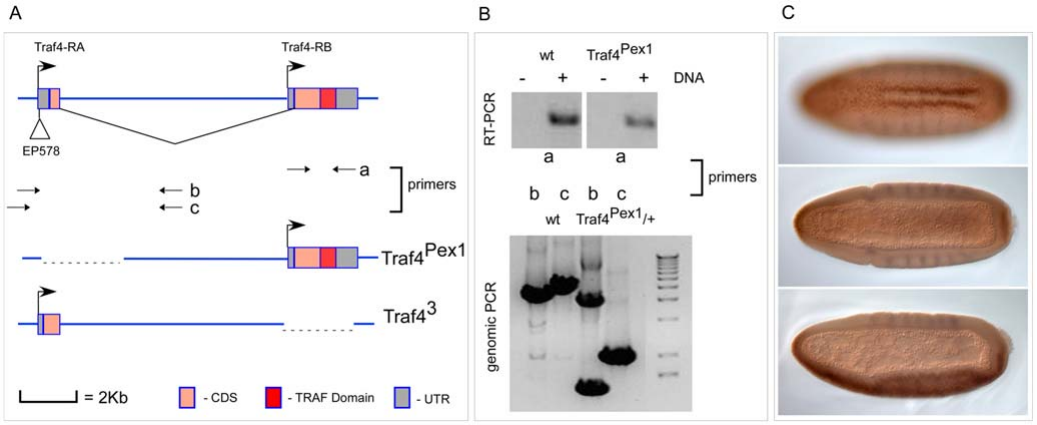
The *traf4* gene, genomic structure of the mutant alleles and the *traf4^3^* mutant phenotype. A. The arrows in the top panel indicate the transcription start sites for the two transcripts (Traf4-RA and Traf4-RB). The insertion site of the P-element EP578 is also shown. The pairs of arrows underneath labelled ‘a’ ‘b’ and ‘c’ indicate the sites of primer pairs used for the PCR analysis of the mutant chromosomes. The mutant chromosomes are in the bottom part of A. The coding region is shown as a pink box, the UTRs as grey boxes, the intron as a line. In the diagrams of the mutant alleles the regions that have been excised (*traf4^Pex1^*) or replaced by the gene disruption vector (*traf4^3^*) are shown as dashed lines. B. The top panel shows the result from RT-PCR performed with primer pair ‘a’ on cDNA from wild type (wt) and *traf4^Pex1^* mutant homozygous animals. The ‘-’ indicates no reverse transcriptase sample to control for DNA contamination and the ‘+’ indicates that reverse transcriptase was used. The bottom panel shows genomic PCR results with primer pairs ‘b’ and ‘c’ on genomic DNA samples from wild type (wt) and traf4^Pex1^ heterozygous animals. Primer pair ‘b’ amplifies both wild type and mutant products in the traf4^Pex1^ heterozygous condition whereas primer pair ‘c’ amplifies only the smaller, mutant product preferentially, possibly because the wild type product size in the case of ‘b’ is smaller compared to ‘c’. C. Ventral (top and middle panels) and lateral (bottom panel) whole mount images of a *traf4^3^* homozygous mutant embryo stained for Twist (brown) and Even skipped (blue) showing the delay in ventral furrow formation.

This region includes the gene *traf4* (formerly designated *traf1*, but changed to *traf4* to reflect its relationship to vertebrate *traf4* [23]) which is expressed in the mesodermal primordium [24]. This suggested *traf4* as a candidate for the gene whose loss may have caused the observed phenotype. To test this we examined whether the expression of Traf4 was able to suppress the defects in the mesoderm of homozygous *Df(2L)ed-dp* embryos. This approach proved difficult because we found that over-expression of Traf4 in the mesoderm of wild type embryos had a dominant effect producing defects similar to those observed in the mutant. We therefore chose to analyse *traf4* loss of function mutants instead. A viable mutant created by insertion of a P-element (*EP578*) had been described as a suppressor of reaper-induced cell death [25], and a small deletion caused by excision of the P-element was reported to have generated a lethal null allele of the *traf4* gene, *traf4* ^ex1^ (formerly *traf1^ex1^* [26]). We found that embryos homozygous for either of these mutations looked normal. However, it was not clear whether the mutations were complete loss of function alleles. Indeed we found that although *traf4* ^ex1^ had been reported to be lethal, it is viable over deficiencies deleting the *traf4* gene, which shows that the reported lethality is not due to loss of Traf4. Furthermore, we find that the mutated gene still allows expression of one of the Traf4 transcripts (*Traf4-RB*) that contains a full open reading frame encoding a protein which includes the Zn-fingers and the MATH domain. The *traf4* gene has two alternative promoters (Figure 6), and while the upstream promoter is deleted in *traf4* ^ex1^, expression from the downstream promoter is unaffected in this mutant. We therefore created a new mutant by targeted homologous recombination [27] which deletes the large Zinc-finger and MATH-domain encoding exon, and which we call *traf4* ^3^ (Figure 6). The chromosome carrying this deletion is lethal in homozygosity and also when heterozygous over deficiencies that delete the *traf4* gene, showing that loss of *traf4* leads to lethality.

*traf4* ^3^ homozygous embryos as well as *traf4* ^3^/*Df(2L)ed-dp* embryos show delays in ventral furrow formation (Figure 6C), indicating that loss of Traf4 is partly or wholly responsible for the initially observed phenotype. A further *traf4* allele, *traf4* ^L2^, (formerly *traf1^L2^* [28]) that became available during this analysis is lethal over our allele and shows the same phenotype. Thus, *traf4* is a gene that is zygotically required for the formation of the ventral furrow.

In summary, we have identified two new loci required for early gastrulation, one acting zygotically in ventral furrow formation, the other a maternal effect gene required for germ band extension. Biochemical and cell biological experiments to test the functions of these genes are now possible.

## Materials and Methods

### Fly stocks and crosses

A list of the fly stocks used in this study and their source is given in Table S1. All stocks were maintained and crosses performed under standard conditions [29], [30], at room temperature, except where otherwise mentioned.

### Generation of Drosdel and Exelixis deletions

Crosses to construct Drosdel deletions were performed according to [13], [14] (originally described in [31]). The deficiencies were confirmed by complementation and PCR. At least five stocks were tested for each deficiency; for each deficiency one of those that did not complement lethal alleles in the region was selected and maintained as a stock. The Exelixis deletion *Df(2L)Exel3006* was made as described in [15].

### Generation of a *traf4* null allele

Targeting for the *traf4* gene was carried out based on the ‘ends out’ targeting strategy [27]. The pW25 vector was used to clone genomic sequences flanking the second exon of the *traf4* gene. For the genomic stretch 5′ to the second exon, the oligos 5′AATGCGGCCGCACTCCAAGGACTGCTCCACTC3′ (Not1 overhang) and 5′AATGGTACCGATTCTGGAGCAAGGGGACTT3′ (Acc65I overhang) were used to amplify an approximately 4 Kb product from genomic DNA and cloned into the same sites in pW25. For the genomic stretch 3′ to the second exon, the oligos 5′AATGGCGCGCCTGAGTCCGAATGTGAGCTTGA3′ (Asc1 overhang) and 5′AATCGTACGCCATTTCTCCAAGGAGGTGAA3′ (BsiW1 overhang) were used to amplify an approximately 4 Kb product from genomic DNA and cloned into the same sites in pW25 to generate the targeting construct. Transgenic flies carrying an insertion of this targeting construct on the 3^rd^ chromosome were used to generate precise targeting events as described [27]. We screened 1400 vials for red eyed flies which are potential homologous recombination events and obtained 15 lines with red eyes which were FLP-insensitive integrations. PCRs with primers designed to amplify precise targeting events were used to confirm specific targeting events. One event was isolated and the allele named *traf4^3^*. Complementation experiments with deficiencies uncovering *traf4* and PCR further confirmed the targeting.

### Embryo fixation and immunohistochemistry

Embryos were fixed and processed according to standard protocols [32].

The primary antibodies used were Rabbit anti-Twist 1∶3000 [17] and Rabbit anti-Eve 1∶5000 [18]. For immunohistochemical detection, a biotinylated secondary antibody (Goat anti-Rabbit from Dianova, Germany) was used and detection was carried out using the Vectastain ABC kit ‘Elite’ (Vector laboratories) using DAB as substrate. 1% cobalt chloride and 1% nickel chloride were added to obtain a blue colour for one of the antibodies in cases where two antigens were detected.

### Mounting, sectioning and imaging of embryos

The whole mount DAB stained embryos were washed in PBS-Tween after staining and dehydrated in an ethanol gradient, followed by incubation in water free acetone. After this step, the embryos were incubated overnight in an acetone: araldite (1∶1) solution at −20°C. Embryos were either stored in this form or mounted on plastic cups (2 cm diameter) where they were sorted, staged and statistically analysed. Individual embryos were mounted in a drop of araldite on glass slides and photographed. Embryos for sectioning were arranged in an araldite block and the block was allowed to polymerize at 50°C overnight. Sections were made on the Leica RM2065 microtome. Imaging was performed using the Zeiss Axioplan microscope and image processing was done using Adobe Photoshop CS2.

### Single embryo PCR

Embryos were collected on an apple juice-agar plates, fixed and stored in methanol. Individual embryos were dispensed in 10 µl of 10 mM Tris-HCl pH 8.2, 1 mM EDTA, 25 mM NaCl and frozen. After a minimum of 30 minutes, the embryos were thawed and 0.2 µl Proteinase K (200 µg/ml stock solution) was added. The embryos were incubated at 37°C for 30 minutes followed by 95°C for 2 minutes for heat inactivation of the Proteinase K. 1 µl of this extract was used as the template for PCR reactions.

The reaction components were: 20pmols of each primer, 10 mM dNTP, 2.5 µl 10X PCR buffer without Mg^2+^(Roche), 2.5 mM Mg^2+^ and 0.25 µl of High Fidelity Taq Polymerase (Expand High Fidelity PCR system from Roche) in a 25 µl PCR reaction mix. The PCR programme included a denaturation step of 3 minutes at 94°C followed by 35 cycles: 30 seconds at 94°C, 1 minute at 50°C the annealing temperature, 3 minutes at 72°C the extension temperature and ending with a final extension of 10 minutes at 72°C.

A list of the primers used for single embryo PCR and their sequences is given in Table S2.

## Supporting Information

Table S1 Description of the stocks used in this study.
(0.17 MB DOC)

Table S2 The genes and the primer sequences used in single embryo PCR.
(0.05 MB DOC)

Figure S1 Representative single embryo PCR with primers for dumpy (dp), N-Cadherin (NCad) CG15631, CG11929, CG15436 and CG3225 on embryos from the Df(2L)dp-h28 (A) and Df(2L)dp-h24 stocks (B). NCad is the positive control, dp, CG11929, CG15631 and CG3225 the candidate genes tested. In embryos 4, 9 and 10 from the Df(2L)dp-h28 stock the NCad primers yield a product of the predicted size, as do dp and CG3225 but the bands for CG15631 are missing. In Df(2L)dp-h24 embryos 5, 6 and 10, the bands for CG15631 and CG15436 are missing. See legend for Table1 for comments.
(0.62 MB TIF)

Figure S2 Schematic representing chromosomal segregation in a compound autosomal stock (A) and the chromosomal combinations in a cross between a second chromosome compound females and a second chromosome deficiency stock (B) (adapted from [1], [20]). The blue and red lines represent the second chromosome left and right arms, and the black spot is the centromere. A. Unlike normal chromosomes where one left arm is joined to one right arm by the centromere, in the compound autosomal stock the two left arms are attached to each other as are the two right arms. These individuals contain the normal genetic complement. However, their gametes contain either two left or two right arms of the attached autosome or all four arms or none. Segregational analysis from previous studies indicate that virtually all the female gametes are either attached right arms or attached left arms whereas male gametes in addition include the category of all four arms attached to each other as well as none. The boxes marked in grey indicate the progeny from the stock that have the full chromosomal complement and maintain the stock. The boxes marked in green are the ones lacking entire right arms or left arms, useful in studying zygotic gene function. B. The broken blue line indicates the deletion in the left arm of the deficiency stock. The bottom left progeny is of interest (shaded in grey), as it has only one second left arm, which is deleted for the region of interest. A gene with zygotic requirement mapping to the region uncovered by the deficiency will show its phenotype as the embryo lacks either copy of the gene but maternal effects of the gene will not show up, as the mothers have a normal chromosomal complement.
(1.48 MB TIF)

Figure S3 Schematic showing the chromosome segregation pattern in a cross between Df(2L)ed-dp, snail recombinant males and Df(2L)ed-dp females (A) and in a cross between second chromosome translocation stock males and Df(2L)ed-dp females (B). The left arm of the second chromosome is shown in blue, the right arm in red. A. The deficient region in the case of the Df(2L)ed-dp chromosome is represented by the purple transverse line, the snail mutation by the green transverse line. These two chromosomes have been recombined together so as to have the chromosome deficient for both Df(2L)ed-dp as well as snail (shown as a chromosome with the purple as well as the green transverse lines). Males carrying such a recombined second chromosome, when crossed to Df(2L)ed-dp females yield progeny with the four chromosomal combinations represented in the figure. The subset of progeny marked in grey (top right box) is the one of interest; the snail gene is present only in one copy while the genomic region uncovered by Df(2L)ed-dp is missing on both chromosomes. B. In the case of deficiency stocks, the left arm has a break, indicating the chromosomal deletion. In the case of the translocation, the two green lines separating the break indicate the translocation. One eighth of embryos from such a cross lack the translocated part of the second left chromosome including the region uncovered by the deficiency and the homologous chromosome is the deficiency chromosome (top right column marked in grey). An enhancement of the zygotic phenotype by haploinsufficiency of loci uncovered by the translocation should be obvious in one eighth of the progeny from such a cross.
(1.32 MB TIF)
