# Supplementary material for: A Small Genomic Region Containing Several Loci Required for Gastrulation in Drosophila
Source: PLoS One. 2009 Oct 13;4(10):e7437. doi: 10.1371/journal.pone.0007437 (PMC2758545; doi:10.1371/journal.pone.0007437)
Supplement: Table S1 — Description of the stocks used in this study. (0.17 MB DOC) [file pone.0007437.s001.doc]

Supporting Table S1: Description of the stocks used in this study.

| **Stock Genotype** | **Stock No./Source** | **Original Reference** |
| --- | --- | --- |
|  |  |  |
| **Deficiencies 24-25 cytogenetic region** |  |  |
| *Df(2L) M24 F-B/SM1* | Bl-744 | Szidonya and Reuter, 1988 |
| *Df(2L) sc 19-11/In(2L) Cy,Roi* | Sz | Szidonya and Reuter, 1988 |
| *Df(2L)dp-h25/In(2L)Cy[L]t[R], In(2R)Cy, amos[Roi-1]* | Bl-3081 | Szidonya and Reuter, 1988 |
| *Df(2L)dp-h19/SM1* | Sz | Szidonya and Reuter, 1988 |
| *Df(2L)dp-h28/SM1* | Sz | Szidonya and Reuter, 1988 |
| *Df(2L)M24F11/Dp(2;2)B3, ed1 dpo2 cl1* | Bl-3080 | Szidonya and Reuter, 1988 |
| *Df(2L) sc 19-11/In(2L) Cy,Roi* | Sz | Szidonya and Reuter, 1988 |
| *Df(2L)sc19-9/In(2L)CyLtR In(2R)Cy, Cy1 amosRoi-1* | Bl-3815 | Szidonya and Reuter, 1988 |
| *Df(2L)sc19-8/SM6b; Dp(2;1)B19, y1, ed1 dpo2 cl1* | Bl-693 | Szidonya and Reuter, 1988 |
| *Df(2L)sc19-4/In(2L)CyLtR In(2R)Cy, Cy1 amosRoi-1* | Bl-3813 | Szidonya and Reuter, 1988 |
| *Df(2L)sc19-3/In(2L)CyLtR In(2R)Cy, Cy1 amosRoi-1* | Bl-3812 | Szidonya and Reuter, 1988 |
| *Df(2L)sc19-7/In(2L)CyLtR In(2R)Cy, Cy1 amosRoi-1* | Bl-3814 | Szidonya and Reuter, 1988 |
| *Df(2L)ed1/CyO; P{ry+t7.2=ftz/lacC}1* | Bl-5330 | Szidonya and Reuter, 1988 |
| *Df(2L)dp-h24/SM6b* | Bl-1070 | Szidonya and Reuter, 1988 |
| *Df(2L) sc 19-10/In(2L) Cy,Roi* | Sz | Szidonya and Reuter, 1988 |
| *Df(2L) ed dp/SM1* | Bl-702 | Szidonya and Reuter, 1988 |
| *Df(2L) sc 19-5/In(2L) Cy,Roi* | Sz | Szidonya and Reuter, 1988 |
| *Df(2L)sc19-1/SM6b; Dp(2;1)B19* | Bl-615 | Szidonya and Reuter, 1988 |
| *Df(2L)dp-cl-h3/Dp(2;2)B3, ed1 dpo2 cl1* | Bl-1185 | Szidonya and Reuter, 1988 |
|  |  |  |
| **Drosdel P element stocks** |  |  |
| *y w iso; P{FRT, w+}RS5-SZ-4048; 3iso* | Drosdel | Ryder *et al*., 2004 |
| *y w iso; P{FRT, w+}RS5-SZ-3590; 3iso* | Drosdel | Ryder *et al*., 2004 |
| *y w iso; P{FRT, w+}RSCB-0544-3; 3iso* | Drosdel | Ryder *et al*., 2004 |
| *y w iso; P{FRT, w+}RS5-HA-1531; 3iso* | Drosdel | Ryder *et al*., 2004 |
| *y w iso; P{FRT, w+}RSCB-5668-3; 3iso* | Drosdel | Ryder *et al*., 2004 |
| *y w iso; P{FRT, w+}RS5-HA-1707; 3iso* | Drosdel | Ryder *et al*., 2004 |
| *y w iso; P{FRT, w+}RSCB-5717-3; 3iso* | Drosdel | Ryder *et al*., 2004 |
| *y w iso; P{FRT, w+}RSUM-8380-3; 3iso* | Drosdel | Ryder *et al*., 2004 |
| *y w iso; P{FRT, w+}RSCB-0621-3; 3iso* | Drosdel | Ryder *et al*., 2004 |
| *y w iso; P{FRT, w+}RS5-HA-1621; 3iso* | Drosdel | Ryder *et al*., 2004 |
| *y w iso; P{FRT, w+}RS5-HA-1035; 3iso* | Drosdel | Ryder *et al*., 2004 |
| *y w iso; P{FRT, w+}RS5-HA-1043; 3iso* | Drosdel | Ryder *et al*., 2004 |
| *y w iso; P{FRT, w+}RSCB-0110-3; 3iso* | Drosdel | Ryder *et al*., 2004 |
| *y w iso; P{FRT, w+}RSCB-0383-3; 3iso* | Drosdel | Ryder *et al*., 2004 |
| *y w iso; P{FRT, w+}RS5-HA-1420; 3iso* | Drosdel | Ryder *et al*., 2004 |
| *y w iso; P{FRT, w+}RS5-SZ-3156; 3iso* | Drosdel | Ryder *et al*., 2004 |
| *y w iso; P{FRT, w+}RSCB-0211-3; 3iso* | Drosdel | Ryder *et al*., 2004 |
| *y w iso; P{FRT, w+}RSCB-0494-3; 3iso* | Drosdel | Ryder *et al*., 2004 |
|  |  |  |
| **Isogenized stocks for generating deletions** |  |  |
| *y w 70FLPiso; Sco/SM6a; 3iso* | Bl6416 | Ryder *et al*., 2004 |
| *w1118 iso; Sco/SM6a; 3iso* | Bl5907 | Ryder *et al*., 2004 |
|  |  |  |
| **Drosdel deletions constructed** |  |  |
| *P{FRT, w+}CB-0621-3r--5-HA-1621r/SM1* | ED260 | unpublished |
| *P{FRT, w+}CB-0211-3r--5-HA-1621r/SM1* | ED256 | unpublished |
| *P{FRT, w+}CB-0494-3r--5-HA-1621r/SM1* | ED258 | unpublished |
| *P{FRT, w+}CB-0383-3r--5-SZ-4048r/SM1* | ED247 | unpublished |
| *P{FRT, w+}CB-5668-3r--5-HA-1531r/SM1* | ED250 | unpublished |
| *P{FRT, w+}CB-5668-3r--5-HA-1035r/SM1* | ED250A | unpublished |
| *P{FRT, w+}CB-5668-3r--5-SZ-3156r/SM1* | ED251 | unpublished |
| *P{FRT, w+}CB-5668-3r--5-HA-1043r/SM1* | ED262 | unpublished |
| *P{FRT, w+}CB-0110-3r--5-HA-1707r/SM1* | ED252 | unpublished |
| *P{FRT, w+}CB-0544-3-3r--5-HA-1420r/SM1* | ED270 | unpublished |
|  |  |  |
| **Exelixis P element insertions** |  |  |
| [PBac{WH}CG15626[f03581]](http://flybase.org/reports/FBti0042250.html) | Bl18671 | Thibault et al 2004 |
| PBac{RB}e03006 | ED252 | Thibault et al 2004 |
| *Exelixis deficiencies* |  |  |
| *Df(2L)Exel6010* | Bl7496 | Parks et al 2004 |
| *Df(2L)Exel9062* | Bl7792 | Parks et al 2004 |
| *Df(2L)Exel3006* |  | unpublished |
|  |  |  |
| **Translocation stocks** |  |  |
| *T(Y;2)A183, y[+]/SM1; C(1)RM, y[1]/C(1;Y)1,y[1]* | Bl2628 |  |
| *T(Y;2)B184, B[S], y[+]/SM1; C(1)RM, y[1]/C(1;Y)1,y[1]* | Bl2645 |  |
| *T(Y;2)G100, y[+]/SM1; C(1)RM, y[1]/C(1;Y)1,y[1]* | Bl2685 |  |
| *T(Y;2)L110, B[S], y[+]/SM1; C(1)RM, y[1]/C(1;Y)1,y[1]* | Bl2739 |  |
| *T(Y;2)H158,y[+]/SM1; C(1)RM, y[1]/C(1;Y)1,y[1]* | Bl3683 |  |
| *T(Y;2)B110, B[S], y[+]/SM1; C(1)RM, y[1]/C(1;Y)1,y[1]* | Bl2640 |  |
| *T(Y;2)B190, y[+]/SM1; C(1)RM, y[1]/C(1;Y)1,y[1]* | Bl2647 |  |
| *T(Y;2)B251, B[S], y[+]/SM1; C(1)RM, y[1]/C(1;Y)1,y[1]* | Bl2661 |  |
| *T(Y;2)J30, y[+]/SM1; C(1)RM, y[1]/C(1;Y)1,y[1]* | Bl2711 |  |
| *T(Y;2)R50, B[S], y[+]/SM1; C(1)RM, y[1]/C(1;Y)1,y[1]* | Bl2763 |  |
| *T(Y;2)B24, y[+]/SM1; C(1)RM, y[1]/C(1;Y)1,y[1]* | Bl2631 |  |
| *T(Y;2)B63, B[S], y[+]/SM1; C(1)RM, y[1]/C(1;Y)1,y[1]* | Bl2633 |  |
| *T(Y;2)B177, B[S], y[+]/SM1; C(1)RM, y[1]/C(1;Y)1,y[1]* | Bl2644 |  |
| *T(Y;2)B238, B[S], y[+]/SM1; C(1)RM, y[1]/C(1;Y)1,y[1]* | Bl2659 |  |
| *T(Y;2)B26, B[S], y[+]/SM1; C(1)RM, y[1]/C(1;Y)1,y[1]* | Bl3682 |  |
| *Tp(2;Y)G/b[1]pr[1]tk[1]* | Bl4359 |  |
|  |  |  |
| **Twist rescue construct** |  |  |
| *Df(2L)TE116(R)GW11Df(2R)S60/SM1* | Rolf Reuter lab | Reuter and Leptin, 1994 |
|  |  |  |
| **Compound stock** |  |  |
| *C(2)v* | Wieschaus lab | Merrill *et al*., 1988 |
|  |  |  |
| **Stocks used for complementation and phenotypic analysis** |  |  |
| *cn^1^ P{ry+t7.2=PZ}l(2)06708^06708^/CyO;ry^506^* | Bl12320 | BDGP |
| *y^1^ w^67c23^; P{w+mC=lacW}ed^k01102^/CyO* | Bl10490 | BDGP |
| *l(2)SH0479/CyO (CG3714)* | Steven Hou lab | Oh et al., 2003 |
| *ft[G-rv]/SM5* | Bl1894 | Bryant et al., 1988 |
| *y^1^ w^67c23^; P{w+mC=lacW}tutl^k14703^/CyO* | Bl10451 | BDGP |
| *l(2)SH0805/CyO (CG18013)* | Steven Hou lab | Oh et al., 2003 |
| *y^1^ w^67c23^; P{w+mC=lacW}Tps1^k08903^/CyO* | Bl10838 | BDGP |
| *dw-24E1 l(2)cg1 ^cg1^/SM5* | Bl292 | Curry, 1941 |
| *y^1^; P{y+mDint2 wBR.E.BR=SUPor-P}mRpL27^KG01128^/SM6a; ry^506^* | Bl14881 | BDGP |
| *l(2)SH0840/CyO (CG15442-RpL27A)* | Steven Hou lab | Oh et al., 2003 |
| *dplv^1^ b^1^/SM5* | Bl278 | Grace, 1980 |
| *l(2)SH1525/CyO (CG2937-mRpS2)* | Steven Hou lab | Oh et al., 2003 |
| *slf^1^ cn^1^ bw^1^ sp^1^/CyO* | Bl3265 | Nusslein-Volhard et al., 1984 |
| *y^1^ w^67c23^; P{w+mC=lacW}l(2)k10004^k10004^/CyO* | Bl10964 | BDGP |
| *y^1^ w^67c23^; P{w+mC=lacW}l(2)k10217^k10217^/CyO* | Bl10983 | BDGP |
| *y^1^ w^67c23^; P{w+mC=lacW}vkg^k00236^/CyO* | Bl10473 | BDGP |
| *y^1^ w^67c23^; P{w+mC=lacW}l(2)k10127^k10127^/CyO* | Bl10973 | BDGP |
| *y^1^ w^67c23^; P{w+mC=lacW}l(2)k11206^k11206^/CyO* | Bl11017 | BDGP |
|  |  |  |
| **EMS alleles in 24-25 cytogenetic region** |  |  |
| *l(2)jf2^a6^/ In(2L)CyLtR In(2R)Cy, Cy^1^ amos^Roi-1^* | Sz | Szidonya and Reuter, 1988 |
| *l(2)jf2^b8^/ In(2L)CyLtR In(2R)Cy, Cy^1^ amos^Roi-1^* | Sz | Szidonya and Reuter, 1988 |
| *l(2)jf3^b25^/ In(2L)CyLtR In(2R)Cy, Cy^1^ amos^Roi-1^* | Sz | Szidonya and Reuter, 1988 |
| *l(2)jf3^sz11^/ In(2L)CyLtR In(2R)Cy, Cy^1^ amos^Roi-1^* | Sz | Szidonya and Reuter, 1988 |
| *l(2)jf3^sz49^/ In(2L)CyLtR In(2R)Cy, Cy^1^ amos^Roi-1^* | Sz | Szidonya and Reuter, 1988 |
| *l(2)jf3^sz56^/ In(2L)CyLtR In(2R)Cy, Cy^1^ amos^Roi-1^* | Sz | Szidonya and Reuter, 1988 |
| *l(2)jf4^b11^/ In(2L)CyLtR In(2R)Cy, Cy^1^ amos^Roi-1^* | Sz | Szidonya and Reuter, 1988 |
| *l(2)jf5^a18^/ In(2L)CyLtR In(2R)Cy, Cy^1^ amos^Roi-1^* | Sz | Szidonya and Reuter, 1988 |
| *l(2)jf5^a19^/ In(2L)CyLtR In(2R)Cy, Cy^1^ amos^Roi-1^* | Sz | Szidonya and Reuter, 1988 |
| *l(2)jf5^b2^/ In(2L)CyLtR In(2R)Cy, Cy^1^ amos^Roi-1^* | Sz | Szidonya and Reuter, 1988 |
| *l(2)jf5^h10^/ In(2L)CyLtR In(2R)Cy, Cy^1^ amos^Roi-1^* | Sz | Szidonya and Reuter, 1988 |
| *l(2)jf5^sz31^/ In(2L)CyLtR In(2R)Cy, Cy^1^ amos^Roi-1^* | Sz | Szidonya and Reuter, 1988 |
| *l(2)jf6^sz3^/ In(2L)CyLtR In(2R)Cy, Cy^1^ amos^Roi-1^* | Sz | Szidonya and Reuter, 1988 |
| *l(2)jf7^h32^/ In(2L)CyLtR In(2R)Cy, Cy^1^ amos^Roi-1^* | Sz | Szidonya and Reuter, 1988 |
| *l(2)jf7^h36^/ In(2L)CyLtR In(2R)Cy, Cy^1^ amos^Roi-1^* | Sz | Szidonya and Reuter, 1988 |
| *l(2)jf7^h39^/ In(2L)CyLtR In(2R)Cy, Cy^1^ amos^Roi-1^* | Sz | Szidonya and Reuter, 1988 |
| *l(2)jf13^sz18^/ In(2L)CyLtR In(2R)Cy, Cy^1^ amos^Roi-1^* | Sz | Szidonya and Reuter, 1988 |
| *l(2)jf14^h7^/ In(2L)CyLtR In(2R)Cy, Cy^1^ amos^Roi-1^* | Sz | Szidonya and Reuter, 1988 |
| *l(2)jf15^h12^/ In(2L)CyLtR In(2R)Cy, Cy^1^ amos^Roi-1^* | Sz | Szidonya and Reuter, 1988 |

Bl – Bloomington

Sz – Szeged stock center
