# Supplementary material for: A Small Genomic Region Containing Several Loci Required for Gastrulation in Drosophila
Source: PLoS One. 2009 Oct 13;4(10):e7437. doi: 10.1371/journal.pone.0007437 (PMC2758545; doi:10.1371/journal.pone.0007437)
Supplement: Table S2 — The genes and the primer sequences used in single embryo PCR. (0.05 MB DOC) [file pone.0007437.s002.doc]

Supporting Table S2: The genes and the primer sequences used in single embryo PCR .

| **Oligo Name** | **Sequence** |
| --- | --- |
| dp1F | GCAATTACACATGCTCCTG |
| dp1R | GATTATCCTGACACTCGTTG |
| CG11929F | CTGGTCTACAATGCAGTAG |
| CG11929R | CAACCAAGCTACTAGTGAC |
| CG15631F | GGAATCAAACAGCTTCACG |
| CG15631F | CAGTTACACTCCTAGTGTC |
| CG3225F | CTGCTCAAGAAGATCCTTC |
| CG3225R | GATGGTTATGATCTCCTCG |
| CG15634F | GTTCTCCATTCGATGGAAC |
| CG15634R | GACTCTGCAGATTCTTGTTG |
| CG3702F | TCGATAGGTCCTCAACACT |
| CG3702R | TCAGCTCACCGAGCATATT |
| AtetF | ATATGCCGACTAATGCCGT |
| AtetR | TGTCCGCTTAGCGAATCAT |
| CG15429F | ACTATCTCAAGGACGAGGT |
| CG15429R | ATGTCGTCCGTGTAGTAGA |
| CG15431F | TAGCAATCGAGTCCAAGCA |
| CG15431R | AGTCCCAATTCTCGATGCT |
| CG15436F | AATGGCGGAAATATGCCGA |
| CG15436R | TGCTCAGCCGAAAAGTCTT |
| CG12677F | CCACACATTTCTGAAGAGG |
| CG12677R | GCGTTGAGTCAATATCGAG |
| Traf4 3'F | GTCTACATAAAGGTCCTGC |
| Traf4 3'R | GCTGCTCCGAATTTAACAC |
| Traf4 5'F | CTGTATCTGAAACTGAGCC |
| Traf4 5'R | CTCTAGATGGCCCTATTTG |
| CG3652F | CTACTAGATGTTCGAGGAC |
| CG3652R | CTAATCACTAAACGAGGCG |
| Tps1F | CACTGTCAACAAGCACTTC |
| Tps1R | GTAGTCATCGAAATCGTCC |

F-Forward primer

R-Reverse primer
