# Supplementary figures and images for: A Small Genomic Region Containing Several Loci Required for Gastrulation in Drosophila
Source: PLoS One. 2009 Oct 13;4(10):e7437. doi: 10.1371/journal.pone.0007437 (PMC2758545; doi:10.1371/journal.pone.0007437)

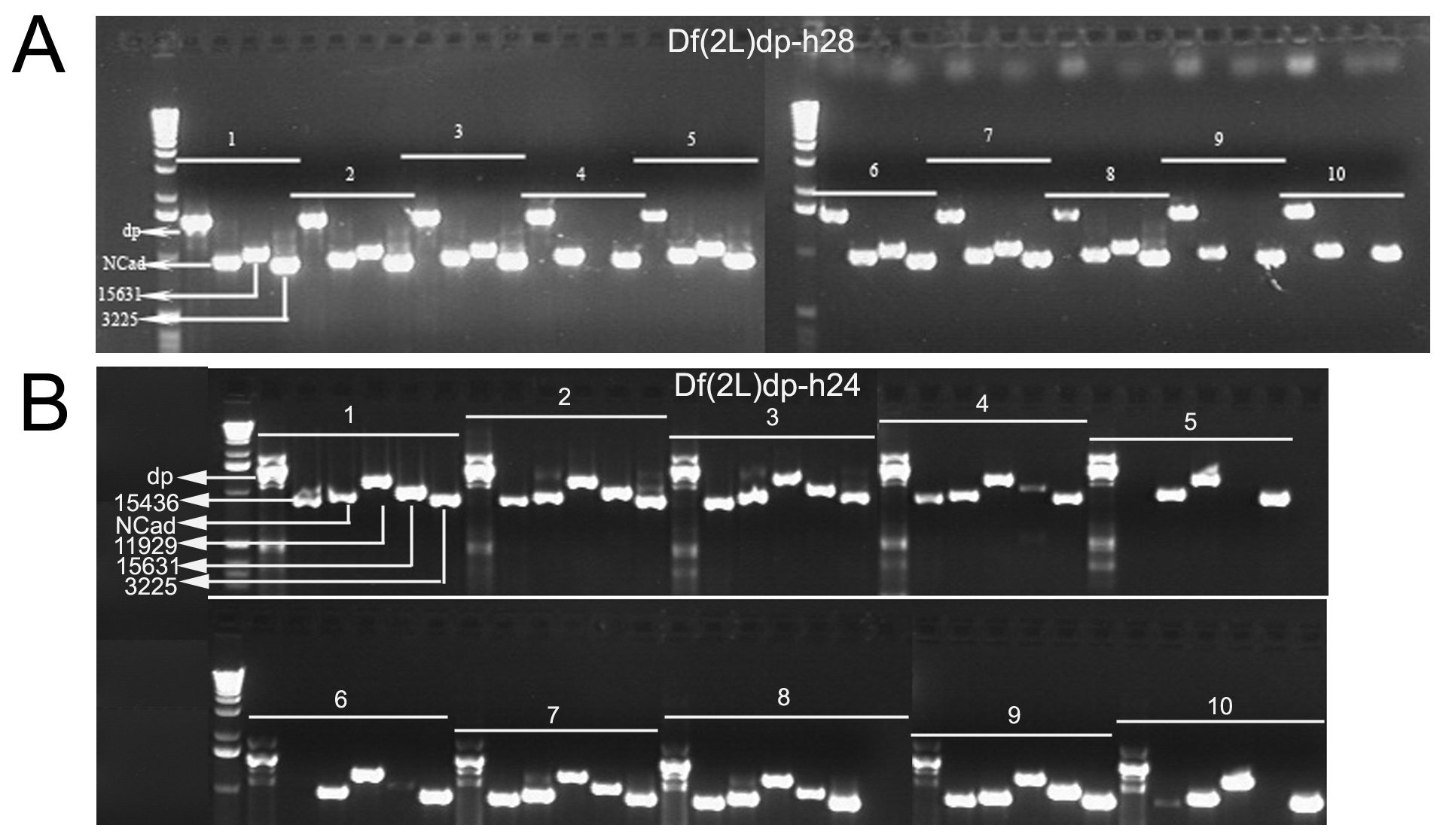

Supplement: Figure S1 — Representative single embryo PCR with primers for dumpy (dp), N-Cadherin (NCad) CG15631, CG11929, CG15436 and CG3225 on embryos from the Df(2L)dp-h28 (A) and Df(2L)dp-h24 stocks (B). NCad is the positive control, dp, CG11929, CG15631 and CG3225 the candidate genes tested. In embryos 4, 9 and 10 from the Df(2L)dp-h28 stock the NCad primers yield a product of the predicted size, as do dp and CG3225 but the bands for CG15631 are missing. In Df(2L)dp-h24 embryos 5, 6 and 10, the bands for CG15631 and CG15436 are missing. See legend for Table1 for comments. (0.62 MB TIF) [file pone.0007437.s003.tif]

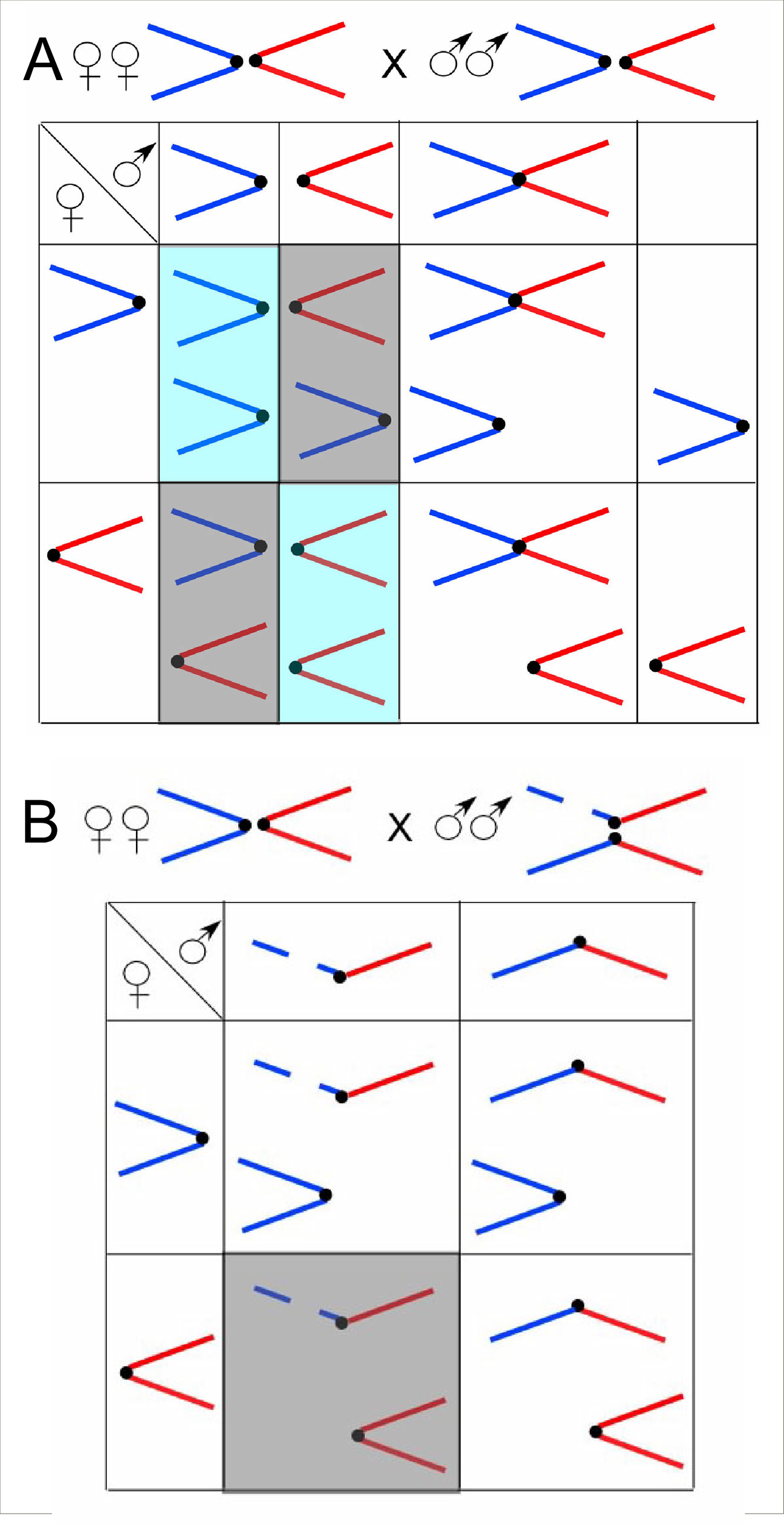

Supplement: Figure S2 — Schematic representing chromosomal segregation in a compound autosomal stock (A) and the chromosomal combinations in a cross between a second chromosome compound females and a second chromosome deficiency stock (B) (adapted from [1], [20]). The blue and red lines represent the second chromosome left and right arms, and the black spot is the centromere. A. Unlike normal chromosomes where one left arm is joined to one right arm by the centromere, in the compound autosomal stock the two left arms are attached to each other as are the two right arms. These individuals contain the normal genetic complement. However, their gametes contain either two left or two right arms of the attached autosome or all four arms or none. Segregational analysis from previous studies indicate that virtually all the female gametes are either attached right arms or attached left arms whereas male gametes in addition include the category of all four arms attached to each other as well as none. The boxes marked in grey indicate the progeny from the stock that have the full chromosomal complement and maintain the stock. The boxes marked in green are the ones lacking entire right arms or left arms, useful in studying zygotic gene function. B. The broken blue line indicates the deletion in the left arm of the deficiency stock. The bottom left progeny is of interest (shaded in grey), as it has only one second left arm, which is deleted for the region of interest. A gene with zygotic requirement mapping to the region uncovered by the deficiency will show its phenotype as the embryo lacks either copy of the gene but maternal effects of the gene will not show up, as the mothers have a normal chromosomal complement. (1.48 MB TIF) [file pone.0007437.s004.tif]

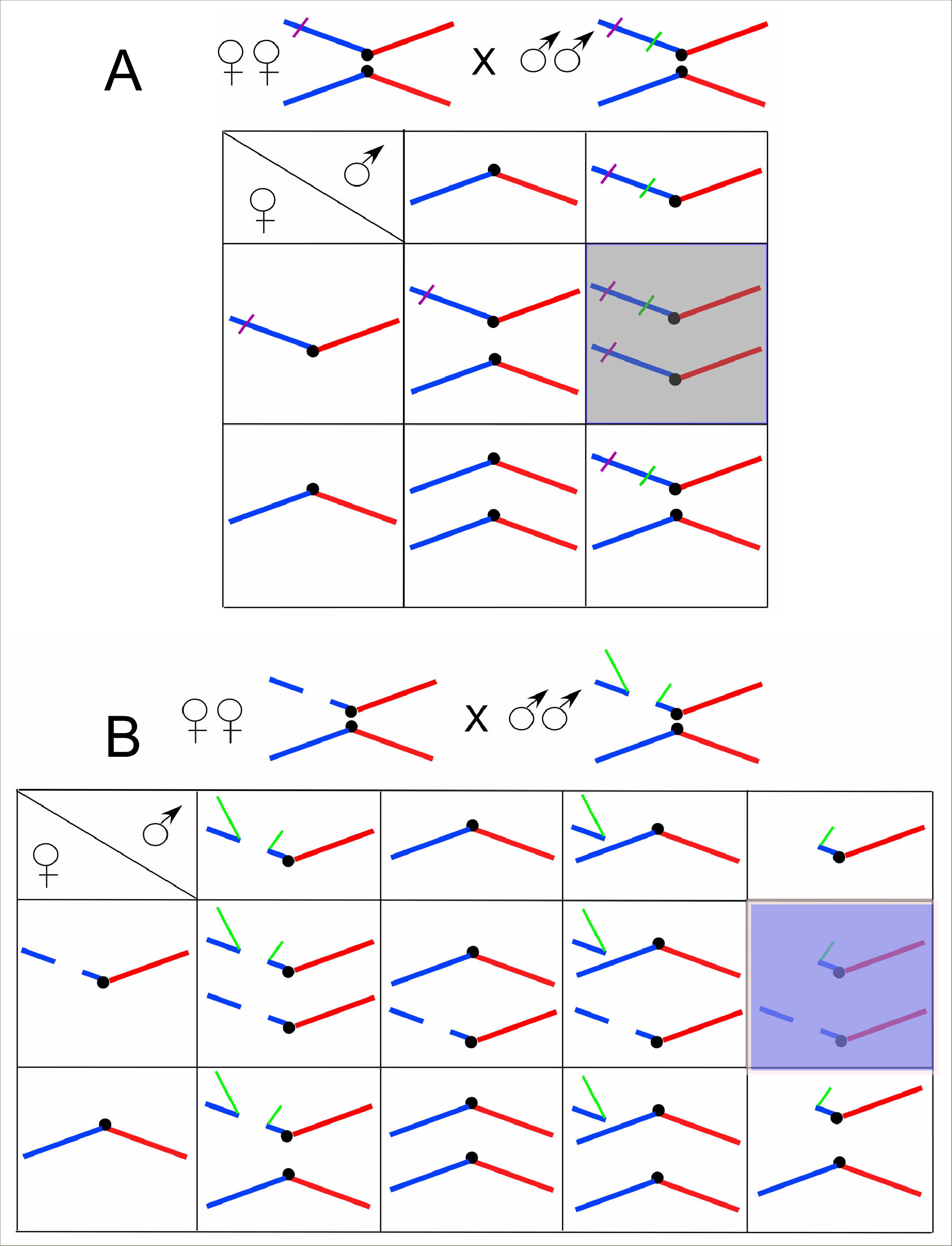

Supplement: Figure S3 — Schematic showing the chromosome segregation pattern in a cross between Df(2L)ed-dp, snail recombinant males and Df(2L)ed-dp females (A) and in a cross between second chromosome translocation stock males and Df(2L)ed-dp females (B). The left arm of the second chromosome is shown in blue, the right arm in red. A. The deficient region in the case of the Df(2L)ed-dp chromosome is represented by the purple transverse line, the snail mutation by the green transverse line. These two chromosomes have been recombined together so as to have the chromosome deficient for both Df(2L)ed-dp as well as snail (shown as a chromosome with the purple as well as the green transverse lines). Males carrying such a recombined second chromosome, when crossed to Df(2L)ed-dp females yield progeny with the four chromosomal combinations represented in the figure. The subset of progeny marked in grey (top right box) is the one of interest; the snail gene is present only in one copy while the genomic region uncovered by Df(2L)ed-dp is missing on both chromosomes. B. In the case of deficiency stocks, the left arm has a break, indicating the chromosomal deletion. In the case of the translocation, the two green lines separating the break indicate the translocation. One eighth of embryos from such a cross lack the translocated part of the second left chromosome including the region uncovered by the deficiency and the homologous chromosome is the deficiency chromosome (top right column marked in grey). An enhancement of the zygotic phenotype by haploinsufficiency of loci uncovered by the translocation should be obvious in one eighth of the progeny from such a cross. (1.32 MB TIF) [file pone.0007437.s005.tif]
